# Circulation of a Meaban-Like Virus in Yellow-Legged Gulls and Seabird Ticks in the Western Mediterranean Basin

**DOI:** 10.1371/journal.pone.0089601

**Published:** 2014-03-13

**Authors:** Audrey Arnal, Elena Gómez-Díaz, Marta Cerdà-Cuéllar, Sylvie Lecollinet, Jessica Pearce-Duvet, Núria Busquets, Ignacio García-Bocanegra, Nonito Pagès, Marion Vittecoq, Abdessalem Hammouda, Boudjéma Samraoui, Romain Garnier, Raül Ramos, Slaheddine Selmi, Jacob González-Solís, Elsa Jourdain, Thierry Boulinier

**Affiliations:** 1 Centre d'Ecologie Fonctionnelle et Evolutive, UMR 5175, CNRS, Montpellier, France; 2 Centre de recherche de la Tour du Valat, Arles, France; 3 Institut de Biologia Evolutiva CSIC-Universitat Pompeu Fabra, Barcelona, Spain; 4 Institut de Recerca i Tecnologia Agroalimentàries (IRTA), Barcelona, Spain; 5 Centre de Recerca en Sanitat Animal (CReSA), Universitat Autònoma de Barcelona (UAB)-Institut de Recerca i Tecnologia Agroalimentaries (IRTA), Bellaterra (Cerdanyola del Vallès), Spain; 6 UMR1161 Virologie INRA, ANSES, ENVA, Animal Health Laboratory ANSES, Maisons-Alfort, France; 7 Institut de Recerca de la Biodiversitat (IRBio), Universitat de Barcelona, Barcelona, Spain; 8 Departament de Biologia Animal, Universitat de Barcelona, Barcelona, Spain; 9 Departamento de Sanidad Animal, Universidad de Córdoba, Córdoba, Spain; 10 Département des Sciences de la Vie, Faculté des Sciences de Gabès, Gabès, Tunisia; 11 Laboratoire de Recherche et de Conservation des Zones Humides, University of Guelma, Guelma, Algeria; 12 Center of Excellence for Research in Biodiversity, King Saud University, Riyadh, Saudi Arabia; 13 INRA, UR346, Unité d'Epidémiologie Animale, Saint Genès Champanelle, France; Thomas Jefferson University, United States of America

## Abstract

In recent years, a number of zoonotic flaviviruses have emerged worldwide, and wild birds serve as their major reservoirs. Epidemiological surveys of bird populations at various geographical scales can clarify key aspects of the eco-epidemiology of these viruses. In this study, we aimed at exploring the presence of flaviviruses in the western Mediterranean by sampling breeding populations of the yellow-legged gull (*Larus michahellis*), a widely distributed, anthropophilic, and abundant seabird species. For 3 years, we sampled eggs from 19 breeding colonies in Spain, France, Algeria, and Tunisia. First, ELISAs were used to determine if the eggs contained antibodies against flaviviruses. Second, neutralization assays were used to identify the specific flaviviruses present. Finally, for colonies in which ELISA-positive eggs had been found, chick serum samples and potential vectors, culicid mosquitoes and soft ticks (*Ornithodoros maritimus*), were collected and analyzed using serology and PCR, respectively. The prevalence of flavivirus-specific antibodies in eggs was highly spatially heterogeneous. In northeastern Spain, on the Medes Islands and in the nearby village of L'Escala, 56% of eggs had antibodies against the flavivirus envelope protein, but were negative for neutralizing antibodies against three common flaviviruses: West Nile, Usutu, and tick-borne encephalitis viruses. Furthermore, little evidence of past flavivirus exposure was obtained for the other colonies. A subset of the *Ornithodoros* ticks from Medes screened for flaviviral RNA tested positive for a virus whose NS5 gene was 95% similar to that of Meaban virus, a flavivirus previously isolated from ticks of *Larus argentatus* in western France. All ELISA-positive samples subsequently tested positive for Meaban virus neutralizing antibodies. This study shows that gulls in the western Mediterranean Basin are exposed to a tick-borne Meaban-like virus, which underscores the need of exploring the spatial and temporal distribution of this flavivirus as well as its potential pathogenicity for animals and humans.

## Introduction

A number of zoonoses are (re)emerging worldwide, the majority of which present risks to animal and human health [1], [2]. Birds are a key animal reservoir since they maintain and are responsible for the large-scale transmission of many infectious diseases [3]–. In recent years, a number of epidemic outbreaks originating in wild birds have been reported, such as outbreaks of West Nile virus or highly pathogenic H5N1 influenza virus, during which the virus later spread to other species of domestic animals and humans [7]–[11]. Thus, surveillance programs targeting wild avifauna and their arthropod vectors are key to thoroughly understanding the eco-epidemiology of many zoonotic diseases.

Arboviruses in the genus *Flavivirus*, including West Nile, dengue, yellow fever, Japanese encephalitis, and tick-borne encephalitis viruses, cause significant disease burdens in humans and animals [12], [13]. Overall, the genus comprises about 70 antigenically related viruses that are geographically widespread [14] and that are mainly classified according to their transmission vector: mosquitoes versus ticks [13]. While most flaviviruses are generally asymptomatic or result in mild illness in humans, they can potentially cause central nervous system diseases, comas, or death [14]. A large variety of flaviviruses are present in Europe [15], [16], especially in the Mediterranean Basin, an area in which a number of bird species reside or stop over [17]. Although several flaviviruses have been found to circulate namely in protected areas in southern Europe, like the wetlands of Camargue [18], [19], Doñana [20], [21], and the Ebro Delta [22], [23], there is nonetheless an urgent need to broaden the surveillance of bird populations, especially those that are close to areas inhabited by humans.

Some flaviviruses are of particular epidemiological concern. West Nile virus (WNV) is widely distributed across Africa, Europe, Australia, and Asia; more recently, it has spread to the Western Hemisphere. Over the last few years, WNV outbreaks have dramatically increased throughout the Mediterranean [24]. Usutu virus (USUV), which is closely phylogenetically related to WNV, has been reported in Spain, Italy, and Central Europe [25], [26], where it has caused significant mortality in captive and wild birds. More recently, a number of studies have revealed that other flaviviruses are circulating in Spain; in particular, an outbreak of the Bagaza virus (BAGV) in 2010 [27] caused an unusually high number of deaths in partridges and, to a lesser extent, in pheasants [27]. These three flaviviruses, WNV, USUV, and BAGV, are transmitted by arthropod vectors, mainly by mosquitoes of the genus *Culex* [16], [28]. However, tick-transmitted flaviviruses, such as tick-borne encephalitis virus (TBEV), which causes fatal neurological infections in humans, are also known to be circulating in Mediterranean countries [15]. In addition, a diverse array of tick-borne flaviviruses (*e.g.*, Meaban virus) is circulating in and among seabird populations. While the pathogenicity of such viruses for birds and humans is still unknown, their potential capacity to spread is of concern, since they may be dispersed over large scales [28]–[30]. Given the potential conservation and epidemiological implications of flavivirus circulation within the Mediterranean, it is important to monitor virus distribution and to assess and predict the risk of disease transmission to humans as well as to domestic and wild animal species.

The yellow-legged gull (*Larus michahellis michahellis*) is a numerically abundant Laridae species [31] that nests in large and dense colonies along the coasts of the Mediterranean Sea [32]. Because the yellow-legged gull is broadly distributed and anthropophilic, it may be more likely to transmit pathogens to humans and domestic animals. This species is also an excellent sentinel species for monitoring flavivirus circulation for several other reasons. First, because yellow-legged gulls form dense colonies and reuse breeding sites year after year, they may be exposed to a number of pathogens and arthropod vectors, such as ticks [33], [34]. Indeed, although non-breeding birds may travel long distances, breeding adults show more restricted movements and high breeding site fidelity [35], which means this long-lived species may facilitate the detection of locally circulating infectious agents. WNV- and USUV-specific antibodies have already been detected in Laridae species in nature [36]–[40]. Experimental infections have also demonstrated that another *Larus* species, the ring-billed gull (*Larus delawarensis*), is susceptible to WNV infection [41]. Second, the yellow-legged gull is potentially involved in the introduction and spread of WNV in the Mediterranean Basin [42]. Based on these observations, we expect that yellow-legged gulls along the western Mediterranean coast are exposed to (1) mosquito-borne flaviviruses that are maintained through enzootic cycles in which wild terrestrial birds act as reservoir hosts, such as WNV [43], USUV [44], or BAGV [27]; and/or (2) tick-borne flaviviruses that are specifically associated with seabirds [13].

Another benefit of using the yellow-legged gull in eco-epidemiological studies is that, in addition to being broadly distributed and numerically abundant, it has been able to colonize a number of habitats, from natural to urban areas and marine to terrestrial ecosystems [45]. As a result, it is also interesting to determine whether yellow-legged gull populations breeding in urban *vs.* coastal areas are more exposed to flaviviruses circulating in terrestrial *vs.* marine habitats [30]. However, exploring the factors that affect the circulation of these viruses at large scales requires extensive sampling and a combination of ecological and biomedical approaches. Here, we implement such an integrative approach by collecting different types of data (*i.e.*, related to gulls and vectors) and extensively sampling breeding sites along the coasts of the western Mediterranean Sea.

Finally, the ability to detect the presence of disease agents in wildlife populations strongly depends on the performance of the various detection methods [46]–[48]. In recent years, immuno-ecological approaches have proven very useful in estimating the probability of species exposure to infectious agents at various temporal and spatial scales; they also provide information about eco-epidemiological processes and help predict disease risk [49]. One such approach takes advantage of the transfer of antibodies from mother to offspring [50]. In birds, adult females that have been exposed to infectious agents can develop humoral immune responses and transfer some of the antibodies produced to the yolk of their eggs [50], [51]. There is a positive correlation between the amount of antibodies in the egg yolk and the amount of antibodies in the plasma of breeding females at the time of laying [52], [53]; therefore, egg yolk antibodies can reflect a female's prior exposure to infectious agents. This indirect method can be useful if the host immune response is detectable for a relatively long time period [53], [54], which has been shown to be the case for WNV in other avian species [55], [56]. Partial clutches can be sampled over a large area that encompasses several host populations, and there is no need to capture breeding adults. Subsequently, the estimates of antibody prevalence obtained with the eggs can establish target locations that should be specifically surveyed in order to characterize the detected infectious agents [53], [54]. The yellow-legged gull offers several advantages in this regard. This gull species has greatly benefited from resources generated by human activities, and, in some areas, it has colonized urban habitats for breeding [57]. Because of this behavior and the negative effect that these gulls have on other bird species, population control efforts, including egg sterilization campaigns, have been implemented around the Mediterranean Basin [32]. As a result, there are no conservation concerns when sampling yellow-legged gull eggs for eco-epidemiological purposes [53], [54].

This study aims to detect the presence of flaviviruses in yellow-legged gulls as well as to provide some initial insights into the eco-epidemiology of flavivirus circulation in the western Mediterranean Basin. First, we screened eggs from yellow-legged gulls across the Mediterranean for flavivirus-specific antibodies. Then, to explore potential vectors in this system, we targeted an area in which flavivirus antibody presence was high; we analyzed nestling sera, soft ticks, and mosquitoes. This strategy enabled us to efficiently focus sampling and analytical efforts on the most relevant target colonies.

## Materials and Methods

### Sampling

**Figure 1:**
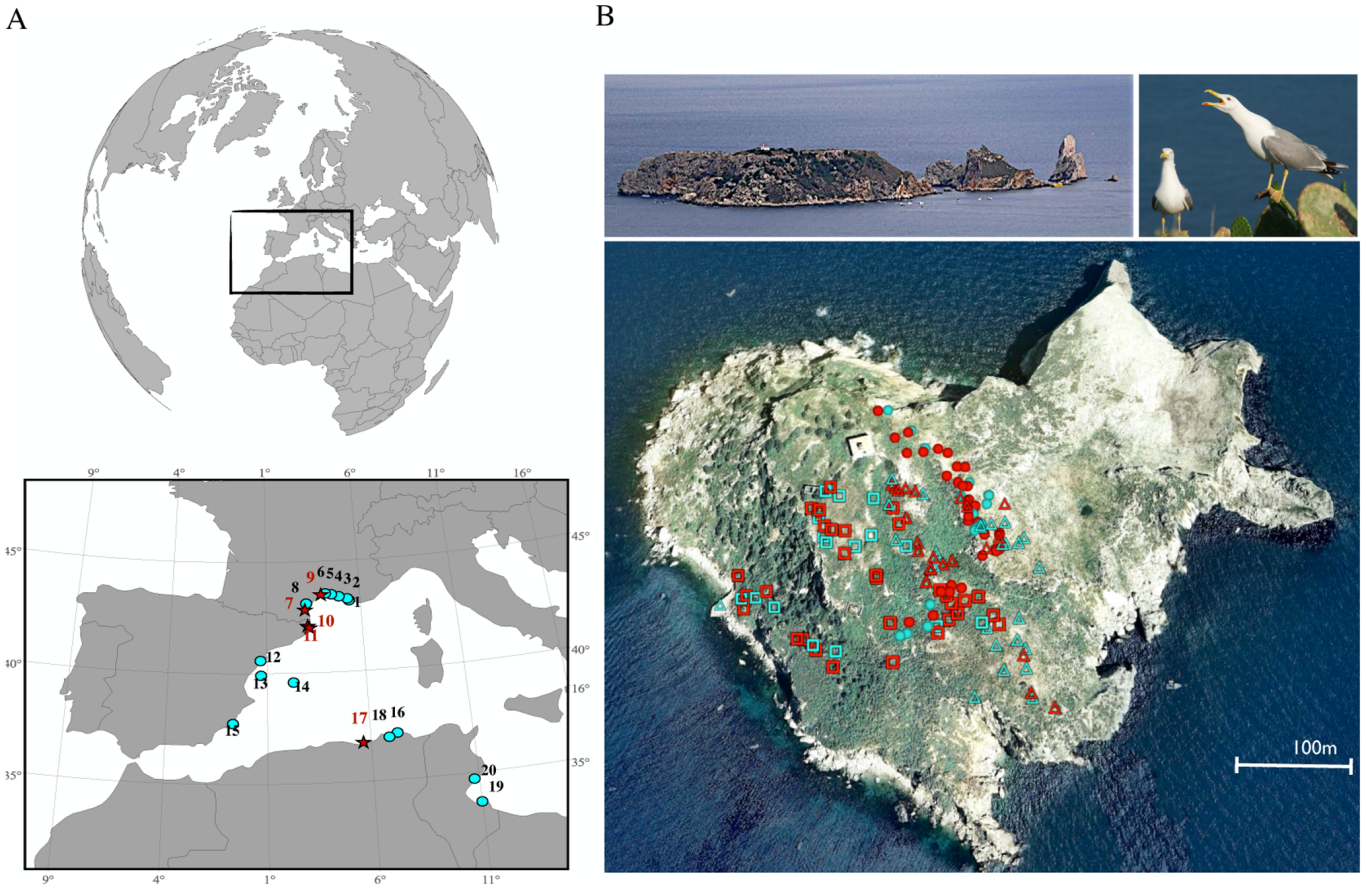
Locations of the colonies sampled in the western Mediterranean (A) and of the nests sampled in the Medes colony (B). (A) Colony numbers are those indicated in Table 1. Red stars indicate colonies that had at least one egg that was positive for antibodies against the flavivirus envelope protein, while turquoise circles indicate colonies that had no positive eggs. (B) The color of a nest indicates its flavivirus-specific antibody status (red: positive, turquoise: negative), and the symbol indicates the sampling year (triangle: 2010, square: 2011, circle: 2012; GPS data were not available for 2009). Doubtful samples were considered to be negative (see text). Pictures by J. González-Solis and taken from Wikimedia commons.

From 2009 to 2011, in March and April, we sampled eggs from 19 breeding colonies located in France (n = 9), Spain (n = 5), Tunisia (n = 2), and Algeria (n = 3) (Figure 1A, Table 1). Ethics committees present at each of the co-authors' institutions specifically approved this study. All efforts were made to minimize animal suffering and disturbance. Briefly, we collected one egg per clutch from nests sampled along randomly selected transects, with the number of transects and their length depending on the spatial configuration of the colony. In smaller colonies, transects were conducted across the whole colony and a majority of nests were sampled, whereas, for larger colonies, transects were performed within a randomly chosen subsection of the colony. In total, 1,098 eggs were analyzed (8–49 eggs per colony, plus 6 eggs from dispersed nests found in the village of L'Escala; Table 1). In some colonies, such as the Medes colony, which is one of the largest yellow-legged gull colonies in the Mediterranean [58], nest locations were recorded using GPS. In the laboratory, the egg yolk was separated from the albumen, homogenized, and frozen at −20°C until analyses took place. Antibodies were extracted from egg yolks using chloroform as previously described [51], and the extract was stored at −20°C.

**Table 1:** Prevalence (%) of antibodies against the flavivirus envelope protein found in yellow-legged gull eggs for all the colonies sampled.

Country	Colony	Coordinates	Year	Prevalence
France	Plane (1)	43°11′14″N-5°23′13″E	2010	0 (0/8)
	Riou (2)	43°10′39″N-5°23′04″E	2009	0 (0/32)
			2010	0 (0/20)
	Frioul (3)	43°16′27″N-5°18′15″E	2009	0 (0/32)
			2010	0 (0/32)
	Carteau (4)	43°22′40″N-4°51′27″E	2009	0 (0/32)
			2010	0 (0/32)
			2011	0 (0/30)
	Besson (5)	43°29′15″N-4°27′47″E	2009	0 (0/32)
			2010	0 (0/32)
	Grau-du-Roi (6)	43°32′15″N-4°08′17″E	2009	0 (0/32)
	Villeneuve (7)	43°30′55.94″-3°54′2.25″E	2009	3 (1/32)
			2010	0 (0/32)
	Gruissan (8)	43°06′36″N-3°06′25″E	2009	0 (0/32)
			2010	0 (0/32)
	Corrège (9)	42°51′15″N-3°01′21″E	2009	3 (1/32)
			2010	3 (1/32)
Spain	L'Escala (10)	42°07′32″N-3°07′58″E	2010	67 (4/6)
	Medes (11)	42°02′50″N-3°13′21″E	2009	37 (14/38)
			2010	49 (24/49)
			2011	67 (33/49)
			2012	67 (34/51)
	Ebro Delta (12)	40°34′17″N-0°39′37″E)	2009	0 (0/32)
			2010	0 (0/29)
			2011	0 (0/30)
	Columbretes (13)	39°53′52″N-0°41′06″E	2010	0 (0/50)
	Dragonera (14)	39°35′00″N-2°19′00″E	2010	0 (0/39)
	Isla Grossa (15)	37°43′40″N-0°42′27″O	2009	0 (0/32)
			2010	0 (0/32)
			2011	0 (0/30)
Algeria	Chetaïbi (16)	37°05′79″N, 7°17′53″E	2009	0 (0/32)
			2010	0 (0/32)
	Jijel (17)	36°47′21″N, 5°36′19″E	2010	3 (1/31)
	Skikda (18)	36°56′15″N, 6°53′09″E	2010	0 (0/17)
Tunisia	Djerba (19)	33°39′10″N-10°58′59″E	2009	0 (0/32)
	Sfax (20)	34°42′28″N-10°45′02″E)	2009	0 (0/32)

The number of positive eggs over the total number of eggs is indicated in parentheses.

Flavivirus antibody prevalence was high in the Medes colony (see Results). As a consequence, additional sampling of eggs (in 2012, n = 51), blood (from 2009 to 2012, n = 501 chicks +2 adult birds), ticks (in 2009, 2011, and 2012, n = 611), and mosquitoes (in 2011, n = 68) was performed. Yellow-legged gull blood (up to 2 mL) was drawn from the tarsal or brachial vein using a sterile syringe. In the lab, the blood was centrifuged at 652 g for 15 min, and the resulting serum was stored at −80°C until analyses took place.

*Ornithodoros maritimus* (soft ticks), which had previously been observed on Medes [33], were sampled from chicks and maintained alive until we returned to the laboratory. A total of 611 ticks were sampled. They were separated into 135 pools (mean pool size of 5), and each pool corresponded to one individual gull chick. In addition, 4 CDC light traps baited with CO_2_ were left running for 24 h in August 2011 in order to capture Culicidae mosquitoes. A total of 68 mosquitoes were sampled, morphologically identified and pooled by species into 10 groups containing up to 13 mosquitoes. The majority of them were identified as *Culex theileri*, a known vector of flaviviruses such as WNV in Europe [16], [59]. Both ticks and mosquitoes were kept frozen at −20°C until analyses took place.

### Serological analyses of eggs and serum

Egg extracts and gull chick serum samples were screened for antibodies directed against the WNV envelope protein, which contains epitopes shared with other viruses of the Japanese encephalitis serocomplex [60]–[62]. We used a commercially available ELISA kit (ID Screen West Nile Competition, ID VET, Montpellier, France) in accordance with the manufacturer's instructions. Results were expressed as a percentage of competition (PC) calculated using the optical density (OD) of a sample and the mean OD of the negative control (NC) of the kit as follows: PC = (OD_sample_/OD_NC_)×100. According to kit instructions, samples with PC values ≤40% were considered positive, those with PC values >50% were considered negative, and those with PC values between 40% and 50% were considered doubtful. In our statistical analyses, doubtful samples were grouped with negative samples.

Virus neutralization tests were used to specifically identify the flaviviruses to which gulls had been exposed [63]. Most of the ELISA-positive eggs (n = 109/113) and gull serum samples (n = 18/18) were screened for neutralizing antibodies against the WNV IS-98-ST1 or Eg101 strains using 96-well plate neutralization tests as previously described, in which 20 was considered to be a positive titer [64]. The presence of neutralizing antibodies against USUV (SAAR-1776 strain) was also assessed using a 96-well plate neutralization test for a subset of the ELISA-positive eggs (n = 14). A plaque reduction neutralization test (PRNT_90_) was also performed on these eggs to detect antibodies against TBEV. A comparable test was performed on ELISA-positive eggs (n = 108) and serum samples (n = 18) to detect antibodies against Meaban virus.

Briefly, the neutralization tests for antibodies against TBEV and Meaban virus were conducted as follows. Six-well plates were seeded with 8×10^5^ Vero (TBEV) or SW13 cells (Meaban virus) per well 1 day before the neutralization assay. Diluted egg extracts (1/20 or 1/40 and 1/100, depending on the available volume) were incubated with TBEV (Hypr strain) or Meaban virus (Brest ART707 strain) in a suspension containing 400 plaque-forming units per mL of Dulbecco's Modified Eagle's Medium (DMEM, Invitrogen, Life Technologies, Saint Aubin, France) for 1h30 in a CO_2_ incubator at 37°C. The culture medium was then removed from the wells, and 0.5 mL of the virus-serum mixture was added to the wells and left for 1h30 at 37°C. One mL gelosa was obtained by mixing equal volumes of carboxymethylcellulose (TBEV; VWR, Fontenay-sous-Bois, France) or Seaplaque agarose (Meaban virus; Lonza, Levallois-Perret, France) with DMEM with 2% fetal calf serum. After 5 days of incubation at 37°C, the cells were rinsed twice, fixed with 4% paraformaldehyde (EMS, Hatfield, PA, USA), and stained with crystal violet (Sigma-Aldrich, Saint-Quentin Fallavier, France) for the purposes of plaque counting. The extract was considered positive if it prevented the formation of viral plaques, *i.e.*, if the number of viral plaques associated with the extract was less than 10% of the number counted in the control well (without serum or egg extract).

### Statistical analyses

Because maternal antibody concentration decreases with age in nestlings [65], the approximate age of each chick was estimated by measuring its bill length. Since bill growth is known to approach linearity from the first to the fifth week, chick age was estimated as follows: age (days) = bill length (mm)*0.963–22.34 (J. González-Solís, unpublished data). Chicks were also weighed to calculate the mass to bill length ratio as a proxy for body condition. To test for differences in age or body condition between infected and non-infected chicks we checked for normality and equality of variances and performed one-way ANOVAs.

We used generalized linear models (GLMs), employing a binomial distribution and logit link, to assess whether flavivirus antibody prevalence in eggs sampled from the Medes colony differed among years. The most parsimonious model was selected using the Akaike information criterion (AIC). Analyses were performed in R 2.12.0 (R Development Core Team, 2010). For each of the three years during which eggs were sampled from the Medes colony, a spatial analysis was conducted to determine whether positive versus negative nests demonstrated spatial structure (Moran's I, with permutation tests for significance; [66]).

### Genetic analyses on the vectors

The 135 tick and 10 mosquito pools were analyzed using a generic reverse-transcription nested polymerase chain reaction (RT-nested PCR) that had been designed to detect the flavivirus ribonucleic acid (RNA) genome [67]. Total RNA was extracted using a QIAamp Viral RNA Mini Kit (Qiagen, Valencia, CA, USA) in accordance with the manufacturer's instructions. Reverse transcription of RNA to cDNA and subsequent amplification were carried out using the Access RT-PCR System (Promega, Madison, WI, USA). A fragment of the viral NS5 gene (143 bp) was amplified using degenerate primers and conditions as previously described [67]. PCR products were electrophoresed on 2% agarose gels and visualized using ethidium bromide. Amplification products were sent away for direct sequencing (Macrogen, Inc.).

Sequence chromatograms were manually verified and then assembled using Geneious v. 5.3.6 (Biomatters Ltd.). We compared the sequences we found to published sequences in GenBank (http://www.ncbi.nlm.nih.gov/) using the basic local alignment search tool (BLAST) to find the best match (E-value≤10^−8^). Mean genetic distances were estimated using MEGA v. 5.2.2 [68]. Then, in order to assign our *NS5* sequences to previously identified flavivirus genospecies (see [69], [70]), we performed a standard phylogenetic analysis using flavivirus sequences amplified from *O. maritimus* together with sequences from a selection of strains representative of the *Flavivirus* genus that are available from GenBank, according to Cook and Holmes [69] (see Table S1). Maximum likelihood (ML) trees were constructed using RAxML GUI (https://sites.google.com/site/raxmlgui/home/files/oldhelp), a graphical front–end tool for RAxML-VI-HPC (randomized axelerated maximum likelihood; [71]); the thorough bootstrap option and 1,000 non-parametric bootstrap replicates were employed. The ML analysis used the general time-reversible (GTR) model, with a gamma model of rate heterogeneity and the invariable sites option; the best-fit model was selected using jModelTest [72]. Trees were visualized using FigTree v. 1.3.1 (http://tree.bio.ed.ac.uk/software/figtree).

## Results

### Antibodies against flaviviruses in yellow-legged gull eggs and chicks

On the Medes Islands and in the nearby village of L'Escala (11 km away), a large proportion (56%; binomial CI_95%_: 49–64) of eggs contained antibodies against the flavivirus envelope protein (Table 1). Conversely, there were almost no ELISA-positive eggs at the other sites sampled (Table 1). Overall, only 4 other eggs were ELISA positive: 1 from the Jijel colony in Algeria in 2010 and 3 from the Corrège (1 in 2009 and 1 in 2010) and Villeneuve (1 in 2009) colonies in France.

Within the Medes colony, the overall proportion of positive nests increased over time; it was 37%, 49%, 67%, and 67% in 2009, 2010, 2011, and 2012, respectively (generalized linear model: slope = 0.4, std error = 0.1, Z value = 3.1, p = 0.002). Furthermore, antibodies against flaviviruses were present in the blood of 16 of the 501 Medes gull chicks (3.19%; binomial CI_95%_: 1.84–5.13; Table 2), as well as in the two adult birds sampled. Neither chick age nor condition differed between positive and negative chicks, whether data from all years were considered (mean ± standard error; negative = 19.75±3.59 days, positive = 21.03±2.71 days, F_1,494_ = 1.98, p = 0.16; negative = 0.0538±0.0105 body condition index, positive = 0.0556±0.0098 BC index, F_1,494_ = 0.48, p = 0.49) or just data from the year with the greatest number of seropositive chicks (in 2012, mean ± standard error; negative = 19.95±3.60 days, positive = 20.75±2.68 days, F_1,242_ = 0.63, p = 0.43; negative = 0.0557±0.0125 body condition index, positive = 0.0576±0.0096 BC index, F_1,242_ = 0.30, p = 0.58). No positive spatial autocorrelation was present in the distribution of ELISA-positive eggs in the colony (results of spatial analysis: p-values>0.05 for Moran's I over all the distance classes considered in the three years analyzed [2010–2012]; Figure 1B).

**Table 2:** Results of virus neutralization tests performed to determine if the anti-flavivirus antibodies found in the egg extracts or chick sera were specific to West Nile (WNV), Usutu (USUV), tick-borne encephalitis (TBEV), or Meaban viruses.

Country	Sample	Colony	Year	ELISA (N)	WNV (N)	USUV (N)	TBEV (N)	Meaban virus (N)
France	Egg extracts	Corrège	2009	1(32)	0 (1)	0 (1)	0 (1)	1 (1)
			2010	1 (32)	0 (1)	0 (1)	0 (1)	-
		Villeneuve	2009	1 (32)	0 (1)	0 (1)	0 (1)	1 (1)
Spain	Egg extracts	L'Escala	2010	4 (6)	0 (2)	0 (2)	0 (2)	2 (2)
		Medes	2009	14 (38)	0 (14)	0 (2)	0 (2)	14 (14)
			2010	24 (49)	0 (22)	0 (3)	0 (4)	22 (22)
			2011	33 (49)	0 (33)	0 (3)	1* (3)	33 (33)
			2012	34 (51)	0 (34)	-	-	34 (34)
	Chick sera	Medes	2009	0 (59)	-	-	-	-
			2010	3 (120)	0 (3)	-	-	3 (3)
			2011	0 (76)	-	-	-	-
			2012	13 (246)	3* (13)	-	-	13 (13)
	Adult sera	Medes	2009	1 (1)	0 (1)	-	-	1 (1)
			2012	1 (1)	0 (1)	-	-	1 (1)
Algeria	Egg extracts	Jijel	2010	1 (31)	0 (1)	0 (1)	-	0 (1)

The numbers indicate the number of positive samples titer values ≥20).

The sample size is indicated in parentheses. The * indicates samples in which titer values were equal to 20 for WNV or TBEV whereas they were ≥20 for Meaban virus.

None of the ELISA-positive eggs contained neutralizing antibodies against WNV, USUV, or TBEV (except for one egg from the Medes Islands, which had a titer value of 20 for TBEV; however, that positive value most likely resulted from a cross-reaction with the Meaban virus as the neutralization titer value of the same sample for Meaban virus was >100; Table 2). In contrast, neutralizing antibodies against Meaban virus were detected in 107 of the 108 ELISA-positive eggs tested (the only negative egg was the one from the Jijel colony in Algeria, see Table 2). Similarly, 16 ELISA-positive chick serum samples contained neutralizing antibodies against Meaban virus, in some cases at high levels (titer values ≥100 for 9 samples; Table S2), but not against WNV (except three chicks with titer values of 20 for WNV and ≥20 for Meaban virus). In addition to the seropositive chicks, two adult birds sampled in 2009 and 2012 contained neutralizing antibodies against Meaban virus; they showed no signs of trauma but exhibited symptoms of illness, such as impaired flight capacity.

### Molecular detection of flaviviruses in the vectors

In the Medes colony, flavivirus RNA was detected in 8 of the 135 tick pools tested (2009: 0/8, 2011: 1/28, and 2012: 7/99). No flavivirus RNA was detected in the mosquitoes. All of the *O. maritimus* flavivirus sequences reported in this article are available from the GenBank database (Accession No. KJ440085-KJ440090). Two of the eight positive tick amplifications resulted in incomplete sequences that were of insufficient quality, and they were not used in subsequent analyses.

**Figure 2:**
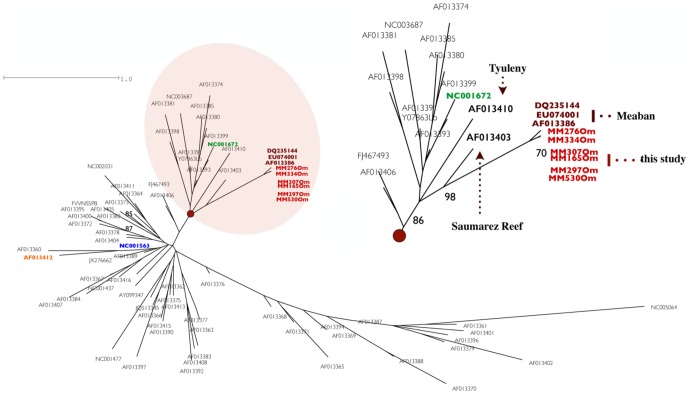
Unrooted maximum-likelihood tree constructed using *NS5* partial gene sequences (143 bp) amplified from *O. maritimus* ticks (red) and a set of sequences from the flavivirus complex (see text). Only bootstrap support values above 70% are indicated above the branches. Reference sequences obtained for viruses belonging to the seabird tick-borne flavivirus group are indicated; these include Meaban (red: this study, dark-red: reference), West Nile (blue), Usutu (orange), and tick-borne encephalitis (green).

The BLAST analysis indicated that the six flavivirus sequences we retrieved significantly matched a *NS5* gene fragment found in Meaban virus (>95% similarity), a flavivirus first isolated in 1981 from *O. maritimus* ticks (Genbank accession number DQ235144 [70]). Phylogenetic analyses showed that our *O. maritimus* flavivirus sequences formed a single well-supported cluster (Bootstrap 70%) that was very closely related to sequences previously identified as Meaban-like (Bootstrap 86%, Figure 2). Mean genetic distance among our *O. maritimus* flavivirus sequences was 0.5%, whereas the mean distance between our sequences and previously published Meaban sequences was 4%. The distance between our Meaban-like sequences and the closest nodes in the phylogenetic tree was 25%; the closest nodes are the Tyuleniy (AF013410) and Saumarez Reef (AF013403) viruses, both of which are tick-borne flaviviruses associated with seabirds. Other flaviviruses, such as WNV, USUV, or TBEV are far from the Meaban-like group in the phylogenetic tree (Figure 2).

## Discussion

### Flavivirus exposure in yellow-legged gulls

Our study is unique in that we used the same protocol to simultaneously survey flavivirus exposure across the western Mediterranean Basin in several breeding colonies of a common wild bird species. Using ELISAs, we found significant differences in flavivirus exposure among yellow-legged gull colonies. In particular, the prevalence of antibodies against the flavivirus envelope protein was high only in the Medes colony.

The high flavivirus-specific antibody prevalence found in the Medes colony and on the few nests sampled from the nearby village of L'Escala (Spain), suggest these are the result of the circulation of a Meaban-like virus, hence a flavivirus closely related to one previously isolated from ticks of *Larus argentatus* in western France (Brittany) [73]. The few positive eggs obtained from the other 19 Mediterranean colonies surveyed, Jijel (Algeria), Villeneuve (France), and Corrège (France), might reflect past flavivirus circulation at those sites. The striking differences observed in prevalence among colonies could be due to a number of factors that are unique to the Medes colony. For example, the Medes colony had the highest density of all the colonies sampled in this study, which might have increased the risk of influx of flavivirus, notably from northern (Bay of Biscay) and southern (North Africa) gull colonies [35], [74]. It is interesting to note that gulls from the Medes colony are in frequent contact with both marine and terrestrial birds, either on their breeding grounds (e.g., European shags *Phalacrocorax aristotelis*) or while feeding on refuse dumps. Constraints could come from the vector; assuming that *Ornithodoros* ticks are the actual vectors of the Meaban-like virus, tick density is relatively large in the Medes colony compared to levels reported in other colonies ([33], authors' pers. obs.). Constraints on tick dispersal among seabird colonies are nevertheless likely to be high [75], even if recent genetic analyses of seabird *Ornithodoros* ticks suggest that long-range tick dispersal by seabirds occurs, at least over long time scales [76]. These different hypotheses cannot be directly tested using the data at hand, but they can still guide future studies in Medes and/or other Mediterranean colonies.

Although antibodies specific for flaviviruses had been reported in three yellow-legged gulls sampled in 2010–2011 in nearby areas [40], our study is the first to report that flaviviruses are circulating in gulls breeding on the Medes Islands and in L'Escala, which is of potential epidemiological relevance as this area is one of the most important recreational hotspots for tourists on the northeastern Mediterranean coast. The fact that the proportion of nests with antibody-positive eggs steadily increased from 2009 to 2012 suggests that at least one flavivirus is currently circulating around Medes. The hypothesis that a flavivirus is currently in circulation is further supported by the fact that flavivirus-specific antibodies were also detected in the serum of Medes yellow-legged gull chicks. If the antibodies of young seropositive chicks were the result of maternal transfer, we would have expected the mean age of seropositive chicks to be lower than that of the seronegative chicks. However, most sampled chicks were over 15 days old and chick age did not differ significantly between positive and negative chicks, which suggests that the detected antibodies were not of maternal origin, particularly given assumptions regarding the rate of decay of maternal antibodies in gull species [65].

The ELISA screening procedure we used detects antibodies against the flavivirus envelope protein, an antigen that is commonly used in WNV serodiagnosis in birds [40], [64], [77]. This protein mediates both receptor-binding and fusion activities after the virus has entered a cell via receptor-mediated endocytosis; because this protein has these crucial functions, it is a useful target for virus-neutralizing antibodies [61]. However, current immune assays that utilize the envelope protein as an antigen have a major drawback: a high degree of cross-reactivity with other antigenically-related flaviviruses [60]. This cross-reactivity can confound the interpretation of serological tests, and it is often impossible to truly identify the virus against which the antibodies in the sample are directed without performing comparative neutralization tests [60], [62], [78]. In this study, neutralization tests showed that the flavivirus-specific antibodies detected by the ELISA did not neutralize WNV, USUV, or TBEV (or did so only poorly: titer value of 20 in one TBE test), which suggests that yellow-legged gulls living in the western Mediterranean Basin have not recently been exposed to these agents. These results are consistent with those obtained by Chastel *et al.* [73] in the late 1970s and early 1980s; they only succeeded in isolating Soldado virus (Nairovirus, Bunyaviridae) from *O. maritimus* ticks found in seabird colonies in southern France and Corsica. However, because WNV has recently been detected in the Mediterranean Basin, notably in Spain [77], [79], [80], North Africa [81], and southern France [18], [43], we could have expected flavivirus antibody prevalence to be high, particularly in the Ebro Delta in Spain [22] or in the Camargue region in France [18]. This prediction was, however, not supported by our results, possibly because the mosquito species that vector WNV do not frequently feed on yellow-legged gulls, and future studies should aim to isolate the virus directly from infected gulls.

### First report of a Meaban-like virus in the Mediterranean

In order to identify the flavivirus or flaviviruses to which yellow-legged gulls in the Medes colony were exposed, potential arthropod vectors were collected and tested to determine if they contained flaviviral RNA. All the mosquito pools tested negative, suggesting that *Culex theileri*, the main mosquito species on Medes, did not play an essential role in transmitting viruses to gulls at the time of sampling. However, the number of mosquitoes tested was very low; therefore, we cannot rule out that this species or other mosquitoes present on Medes may be involved in the flavivirus epidemiological cycle. Conversely, RNA extracts from *O. maritimus* seabird ticks contained flaviviral RNA and, furthermore, the RNA sequence retrieved was more than 95% similar to a fragment of the *NS5* gene of Meaban virus, a seabird tick-borne virus isolated from colonies of a very closely related gull species [82]. The tick *O. maritimus* may infest up to 90% of the gull nests in the Medes colony [83] and is known to affect chick growth [33]. Meaban virus was first isolated from this same seabird tick species, in a sample taken from a herring gull (*Larus argentatus*) colony in Brittany, France [82]. Consequently, Meaban virus neutralization tests were conducted on most of the ELISA-positive egg and serum samples from Medes (Table 2), and all the samples were able to neutralize Meaban virus. This result shows that a significant proportion of the yellow-legged gulls breeding on the Medes Islands had been exposed to a Meaban-like virus. The true seroprevalence may actually be underestimated because we only screened ELISA-positive samples for Meaban virus neutralizing antibodies (*i.e.*, we did not check that the ELISA was more sensitive than the Meaban virus PRNT_90_). Neutralization tests for Meaban virus were likewise positive for the ELISA-positive eggs detected in other colonies in France and Spain (Corrège and Villeneuve, France; L'Escala, Spain) but were negative for Jijel in Algeria (Table 2). This result might reflect the dispersal of a few seropositive female gulls, which subsequently transmitted antibodies to their eggs at their new breeding locations; alternatively, it could suggest that gulls in these colonies are locally exposed to the virus.

*Ornithodoros* ticks have been reported to harbor and transmit a number of disease agents, including numerous flaviviruses [34]. The main species present in the Mediterranean, *O. maritimus*, is known to be abundant in yellow-legged gull colonies, both on Medes as well in some of the other colonies sampled in the present study in which no flavivirus-specific antibodies were detected (*e.g.*, Riou and Frioul, southern France; K.D. McCoy and R. Garnier, *pers. obs.*; [73]). The high local antibody prevalence in the Medes colony may be explained by the fact that infected vectors and viremic adult gulls show restricted movements. Yellow-legged gulls are relatively faithful to their breeding sites [84], but juveniles may move along the Atlantic Coast and around the Mediterranean Basin in the winter [31], [35]. Nevertheless, the dispersal of the Meaban-like virus discovered here is likely to be constrained by the biology of its tick vector, *O. maritimus*, a nidicolous tick species that feeds for only short periods of time on its hosts [34], and by the fact that breeding gulls show limited movement among colonies. However, recent work has suggested that *Ornithodoros* ticks might be able to disperse long distances as a result of the movements of their seabird hosts, although this dispersal may occur only when considered over an evolutionary time scale [76]. Population genetic analyses on the ticks that allow the assessment of contemporary gene flow are now needed to confirm this hypothesis [75].

Despite the potential risks, there is no evidence to date to suggest that this tick species can transmit diseases to humans. This may be explained in part because this tick is seabird specific and its seabird hosts have restricted distributions. However, it has been reported that *Ornithodoros* spp. may bite non-bird species, including humans, when they occur at high infestation densities [34]. Furthermore, the existence of contact zones in certain seabird colonies, where gulls co-occur with other terrestrial vertebrate species and thus more generalist tick vectors like *Ixodes ricinus*, could provide opportunities for pathogen transmission between marine and terrestrial compartments [85]. As a consequence, the epidemiological risk posed by *O. maritimus* to other wildlife species, domestic animals, and, potentially humans, cannot be ruled out entirely. More research should be dedicated to investigating the host range, distribution, and role of *Ornithodoros* species as disease vectors to help clarify the epidemiological risk they pose.

Apart from the risk related to vector driven transmission, gulls may also serve as bridges for the spread of infectious agents. In particular, since yellow-legged gulls are known to colonize urban areas, it is important to consider the possibility that the virus may be dispersed to novel locations [57]. Given that the nests containing antibody-positive eggs were widely dispersed across the Medes colony, the local circulation of the virus is unlikely to be restricted. The antibody-positive eggs found in L'Escala gull nests likely reflect urban colonization by Medes gulls; the village is only 11 kilometers from the islands. It is crucial to understand the implications of the colonization of urban habitats because little is known about the zoonotic potential of Meaban or Meaban-like viruses. Chastel *et al.* [86] did not detect any neutralizing antibodies against Meaban virus in the 562 sera collected from human beings living in Brittany. However, other flaviviruses associated with wild birds are either demonstrated (*e.g.*, WNV, TBEV) [87], [88] or suspected (*e.g.*, Tyuleniy viruses) human pathogens [86].

## Conclusion and Perspectives

In this study, we report the first detection of a Meaban-like virus in the western Mediterranean Basin. Despite widespread sampling, our results suggest that this flavivirus is present at mainly one location: the Medes Islands (Spain). Since antibodies were found in pre-fledging chicks and because of the fact that the prevalence of antibody-positive eggs increased over time in this colony over the three year study period, it would seem that the Meaban-like virus, whose zoonotic potential is unknown, is currently circulating among Medes gulls; isolation trials using ticks and gull serum are currently being conducted to test this hypothesis. In order to better predict the risk of spread of this Meaban-like virus will spread within the Mediterranean Basin, it would be interesting to conduct further investigations that target other bird species that nest on the Medes Islands, as well as to sample the seabird tick *O. maritimus* from additional locations and across different time periods. The host range of this Meaban-like virus also remains to be investigated, particularly since *O. maritimus* ticks occur on other avian species such as European shags [89], which also breed on the Medes Islands. Indeed, tight relationships between seabirds and ticks have important implications for the global epidemiology and evolutionary ecology of zoonotic diseases, as has been previously observed for other tick-borne pathogens; for example, these relationships provide links between marine and terrestrial enzootic transmission cycles [76], [90].

In conclusion, gathering further data on the diversity, distribution, and pathogenicity of viruses associated with seabirds is important, especially given the potential risks the viruses pose for human health. Our study has shown that a combination of population ecology and biomedical approaches can efficiently enhance our understanding of emerging infectious pathogens. Future studies should more thoroughly examine tick vector competence, infection rates, and population genetic structure [90].

### Ethical statement

Ethics committees present at each of the co-authors' institutions specifically approved this study. These include the following: Direction Régionale de l'Environnement, de l'Aménagement et du Logement (DREAL), Syndicat Mixte des Etangs Littoraux (SIEL), Conservatoire Etudes des Ecosystèmes de Provence-Alpes du sud (CEEP), Parc national de Port-Cros, Service Environnement de la Mairie de Gruissan (Aude), Departament d'Agricultura, Ramaderia, Pesca, Alimentació i Medi Natural de la Generalitat de Catalunya, Parc Natural de El Montgrí, les Illes Medes i el Baix Ter, Dirección General de Patrimonio Natural y Biodiversidad de Murcia, Conselleria de Medi Ambient i Mobilitat del Govern de les Illes Balears, Tomás Montalvo (Agència de Salut Pública de Barcelona), Parc Natural del Delta de l'Ebre, Cabildo de Gran Canaria, Conselleria de Medi Ambient, Aigua, Urbanisme i Habitatge de la Generalitat Valenciana, the forest service in the Tunisian Ministry of Agriculture (permit number 518-28/02/2009), and the Algerian DGRSTD/MESRS and DSFP, King Saud University (Saudi Arabia). All efforts were made to minimize animal suffering and disturbance. In the Mediterranean Basin, the yellow-legged gull is a numerically abundant species and is subject to population control measures [34], [36], which facilitates the capture of adults and the sampling of eggs. Sampling of partial clutches early in the breeding season is unlikely to affect local population demography.

## Supporting Information

Table S1 Reference sequences used in the phylogenetic analysis classified according to the flavivirus group sensu Heinz et al. 2001, Cook & Holmes 2001 (see Fig. 2).
(DOCX)

Table S2 Detailed results of Meaban virus neutralization tests performed on ELISA-positive samples. The column ‘Titer >20’ corresponds to samples for which insufficient serum was available to make further dilutions.
(DOC)
