# Supplementary material for: Circulation of a Meaban-Like Virus in Yellow-Legged Gulls and Seabird Ticks in the Western Mediterranean Basin
Source: PLoS One. 2014 Mar 13;9(3):e89601. doi: 10.1371/journal.pone.0089601 (PMC3953012; doi:10.1371/journal.pone.0089601)
Supplement: Table S1 — Reference sequences used in the phylogenetic analysis classified according to the flavivirus group sensu Heinz et al. 2001, Cook & Holmes 2001 (see Fig. 2). (DOCX) [file pone.0089601.s001.docx]

| **Flavivirus** | **Strain** | **Group** | | **NS5 Ac. No** | | | |
| --- | --- | --- | --- | --- | --- | --- | --- |
| Apoi |  | Modoc | | AF013361 | | | |
| Alfuy | MRM-3929 | Japanese encephalitis | | AF013360 | | | |
| Aroa | VenA-1809 | Aroa | | AF013362 | | | |
| Bagaza | DakAr-B209 | Natya | | AF013363 | | | |
| Banzi |  | Yellow fever | | FVVNS5P | | | |
| Batu | P70-1459 | Rio Bravo | | AF013369 | | | |
| Bouboui | DakArB490 | Yellow fever | | AF013364 | | | |
| Bukalasa bat | UGBP-111 | Rio Bravo | | AF013365 | | | |
| Bussuquara | BeAn4073 | Aroa | | AF013366 | | | |
| Cacipacore | BeAn327600 | Japanese encephalitis | | AF013367 | | | |
| Dengue 1 |  | Dengue | | NC_001477 | | | |
| Dengue 2 |  | Dengue | | FJ467493 | | | |
| Dengue 3 | D3/H/IMTSSA-MART/2001/2336 | Dengue | | AY099347 | | | |
| Dengue 4 |  | Dengue | | JQ513345 | | | |
| Kamiti River |  | Unclassified | | NC_005064 | | | |
| Louping ill |  | Louping ill | | Y07863 | | | |
| Meaban |  | Seabird tick-borne | | DQ235144 | | | |
| Meaban | Brest-ART707 | Seabird tick-borne | | AF013386 | | | |
| Meaban | BREST-ART70 | Seabird tick-borne | | EU074001 | | | |
| Carey Island | P70-1215 | Rio Bravo | | AF013368 | | | |
| Cowbone Ridge | W-10986 | Modoc | AF013370 | | | | |
| Dakar bat | 209 | Rio Bravo | AF013371 | | | | |
| Edge Hill | AusC-281 | Yellow fever | AF013372 | | | | |
| Entebbe bat | UgIL-30 | Entebbe bat | AF013373 | | | | |
| Gadgets Gully | CSIRO122 | Mammalian tick-borne | | | AF013374 | | |
| Iguape | SPAn71686 | Aroa | | | AF013375 | | |
| Ilheus |  | Natya | | | AF013376 | | |
| Israel turkey meningoencephalitis |  | Natya | | | AF013377 | | |
| Japanese encephalitis |  | Japanese encephalitis | | NC_001437 | | | |
| Jugra | P9-314 | Yellow fever | | | | AF013378 | |
| Jutiapa | JG-128 | Modoc | | | | AF013379 | |
| Kadam | MP-6640 | Mammalian tick-borne | | | | AF013380 | |
| Karshi | LEIV-2247 | Mammalian tick-borne | | | | AF013381 | |
| Kedougou | DakarD1470 | Dengue | | | | AF013382 | |
| Kokobera | AusMRM32 | Kokobera | | | | AF013383 | |
| Koutango | DakarD1470 | Japanese encephalitis | | | | AF013384 | |
| Kunjin | CH16532 | Japanese encephalitis | | | | | JX276662 |
| Kyasanur forest disease | W371 | Mammalian tick-borne | | | | AF013385 | |
| Modoc | M544 | Modoc | | | | AF013387 | |

| **Flavivirus** | | **Strain** | | **Group** | | | **NS5 Ac. No** |
| --- | --- | --- | --- | --- | --- | --- | --- |
| Montana myotis leukoencephalitis | | 40649 | | Rio Bravo | AF013388 | | |
| Murray Valley | |  | | Japanese encephalitis | AF013389 | | |
| Naranjal | | 25008 | | Aroa | AF013390 | | |
| Negishi | |  | | Tick-borne encephalitis | AF013391 | | |
| Ntaya | |  | | Natya | AF013392 | | |
| Omsk_Kubrin | |  | | Mammalian tick-borne | AF013393 | | |
| Phnom Penh bat | | CAMA-38D | | Rio Bravo | AF013394 | | |
| Potiskum | | IBAN10069 | | Yellow fever | AF013395 | | |
| Powassan |  | | | Mammalian tick-borne | | NC_003687 | |
| Rio Bravo | | M-64 | | Rio Bravo | AF013396 | | |
| Rocio | | H-34675 | Natya | | AF013397 | | |
| Royal Farm | | EgArt371 | Mammalian tick-borne | | AF013398 | | |
| Russian Spring Summer encephalitis | | Sofjin | Tick-borne encephalitis | | AF013399 | | |
| Saboya | | DakAnD4600 | Yellow fever | | AF013400 | | |
| Sal Vieja | | 38TWM-106 | Modoc | | AF013401 | | |
| San Perlita | | 71V-1251 | Modoc | | AF013402 | | |
| Saint Louis encephalitis | | MSI-7 | Japanese encephalitis | | AF013416 | | |
| **Saumarez Reef** | | **CSIRO-4** | **Seabird tick-borne** | | **AF013403** | | |
| Sepik | | MK7148 | Yellow fever | | AF013404 | | |
| Sokuluk | | LEIV-400K | Entebbe bat | | AF013405 | | |
| Spondweni | | SAAR-94 | Spondweni | | AF013406 | | |
| Stratford | | AUSC-338 | Kokobera | | AF013407 | | |
| Tembusu | | MM1775 | Natya | | AF013408 | | |
| Tick borne encephalitis |  | | | Tick-borne encephalitis | | NC_001672 | |
| **Tyuleniy** | | **LEIV-6C** | **Seabird tick-borne** | | **AF013410** | | |
| Uganda S | |  | Yellow fever | | AF013411 | | |
| Usutu | | SAAR-1776 | Japanese encephalitis | | AF013412 | | |
| West Nile | |  | Japanese encephalitis | | NC_001563 | | |
| Yaounde | | DakArY 276 | Japanese encephalitis | | AF013413 | | |
| Yellow fever |  | | | Yellow fever | | NC_002031 | |
| Zika | | MR-766 | Spondweni | | AF013415 | | |
