# Supplementary material for: Circulation of a Meaban-Like Virus in Yellow-Legged Gulls and Seabird Ticks in the Western Mediterranean Basin
Source: PLoS One. 2014 Mar 13;9(3):e89601. doi: 10.1371/journal.pone.0089601 (PMC3953012; doi:10.1371/journal.pone.0089601)
Supplement: Table S2 — Detailed results of Meaban virus neutralization tests performed on ELISA-positive samples. The column ‘Titer >20’ corresponds to samples for which insufficient serum was available to make further dilutions. (DOC) [file pone.0089601.s002.doc]

| **Country** | **Samples** | **Colony** | **Year** | **Negative** | **Titer 20** | **Titer >20** | **Titer 40** | **Titer 100** | **Titer >100** | **Total tested** |
| --- | --- | --- | --- | --- | --- | --- | --- | --- | --- | --- |
| France | Egg extracts | Corrège | 2009 | 0 | 0 | 0 | 0 | 1 | 0 | **1** |
|  |  | Villeneuve | 2009 | 0 | 0 | 0 | 1 | 0 | 0 | **1** |
| Spain | Egg extracts | L’Escala | 2010 | 0 | 1 | 0 | 0 | 0 | 1 | **2** |
|  |  | Medes | 2009 | 0 | 7 | 0 | 1 | 0 | 6 | **14** |
|  |  |  | 2010 | 0 | 5 | 1 | 0 | 1 | 15 | **22** |
|  |  |  | 2011 | 0 | 5 | 0 | 2 | 3 | 23 | **33** |
|  |  |  | 2012 | 0 | 3 | 0 | 2 | 1 | 28 | **34** |
|  | Chick sera | Medes | 2010 | 0 | 0 | 0 | 0 | 0 | 3 | **3** |
|  |  |  | 2012 | 0 | 6 | 1 | 0 | 1 | 5 | **13** |
|  | Adult sera | Medes | 2009 | 0 | 1 | 0 | 0 | 0 | 0 | **1** |
|  |  |  | 2012 | 0 | 0 | 0 | 0 | 0 | 1 | **1** |
| Algeria | Egg extracts | Jijel | 2010 | 1 | 0 | 0 | 0 | 0 | 0 | **1** |
